# Clinical risk conditions for acute lung injury in the intensive care unit and hospital ward: a prospective observational study

**DOI:** 10.1186/cc6113

**Published:** 2007-09-04

**Authors:** Niall D Ferguson, Fernando Frutos-Vivar, Andrés Esteban, Federico Gordo, Teresa Honrubia, Oscar Peñuelas, Alejandro Algora, Gema García, Alejandra Bustos, Inmaculada Rodríguez

**Affiliations:** 1 Interdepartmental Division of Critical Care Medicine, and Department of Medicine, Division of Respirology, University Health Network, University of Toronto, 399 Bathurst Street, F2-150, Toronto, Ontario M5T 2S8, Canada; 2 Intensive Care Unit, Hospital Universitario de Getafe, CIBER de Enfermades Respiratorios, Carretera de Toledo Km 12,500, 28905 Madrid, Spain; 3 Intensive Care Unit, Fundacíon Hospital de Alcorcón, c/Budapest 1, 28922 Alcorcón, Madrid, Spain; 4 Intensive Care Unit, Hospital de Móstoles, c/Río Jucar, 28935 Móstoles, Madrid, Spain

## Abstract

**Background:**

Little is known about the development of acute lung injury outside the intensive care unit. We set out to document the following: the association between predefined clinical conditions and the development of acute lung injury by using the American–European consensus definition; the frequency of lung injury development outside the intensive care unit; and the temporal relationship between antecedent clinical risk conditions, intensive care admission, and diagnosis of lung injury.

**Methods:**

We conducted a 4-month prospective observational study in three Spanish teaching hospitals, enrolling consecutive patients who developed clinical conditions previously linked to lung injury, both inside and outside the intensive care unit. Patients were followed prospectively for outcomes, including the diagnosis of acute lung injury or acute respiratory distress syndrome.

**Results:**

A total 815 patients were identified with at least one clinical insult; the most common were sepsis, pneumonia, and pancreatitis. Pulmonary risk conditions were observed in 30% of cases. Fifty-three patients (6.5%) developed acute lung injury; 33 of these (4.0%) met criteria for acute respiratory distress syndrome. Lung injury occurred most commonly in the setting of sepsis (46/53; 86.7%), but shock (21/59; 36%) and pneumonia (20/211; 9.5%) portended the highest proportional risk; this risk was higher in patients with increasing numbers of clinical risk conditions (2.2%, 14%, and 21% (*P *< 0.001) in patients with one, two, and three conditions, respectively). Median days (interquartile range) from risk condition to diagnosis of lung injury was shorter with pulmonary (0 (0 to 2)) versus extrapulmonary (3 (1 to 5)) (*P *= 0.029) risk conditions. Admission to the intensive care unit was provided to 9/20 (45%) patients with acute lung injury and to 29/33 (88%) of those with acute respiratory distress syndrome. Lung injury patients had higher mortality than others (acute lung injury 25.0%; acute respiratory distress syndrome 45.5%; others 10.3%; *P *< 0.001).

**Conclusion:**

The time course from clinical insult to diagnosis of lung injury was rapid, but may be longer for extrapulmonary cases. Some patients with lung injury receive care and die outside the intensive care unit; this observation needs further study.

## Introduction

Conceptually, acute respiratory distress syndrome (ARDS) is an inflammatory lung injury involving both endothelial and epithelial layers of the alveolar-capillary membrane, with subsequent alveolar flooding and formation of a hyaline membrane, arising either from a direct (pulmonary) or indirect (extrapulmonary) insult [1-6]. In clinical practice and in research studies, this ARDS concept is most commonly captured by using the 1994 American–European Consensus Conference (AECC) definition [3,7-9]. Acute lung injury (ALI) is defined as the acute onset of hypoxemia (PaO_2_/FiO_2 _(partial pressure of arterial oxygen/fractional concentration of inspired oxygen) ≤ 300 mmHg) and bilateral infiltrates on frontal chest X-ray, in the absence of left atrial hypertension. ARDS comprises the severe end of the ALI spectrum, defined with the same criteria, except that the hypoxemia threshold is 200 mmHg [3].

In recent years several multicentre observational studies have examined ARDS epidemiology in terms of incidence, risk factors, and associations with mortality [7,9-16]. All of these studies, however, examined antecedent clinical insults from the perspective of patients with ALI or ARDS, reporting the proportion of cases that were due, for example, to pneumonia or sepsis. Studies examining these associations from the perspective of patients at risk of ALI/ARDS are both less prevalent and less recent, all reporting data collected in the early 1980s [17-19]. Because of the time at which they were performed, none of these studies was able to use current clinical definitions for ALI/ARDS or other clinical entities such as sepsis syndrome [20]. In addition, all of the studies outlined above identified patients who were admitted to an intensive care unit (ICU) [7,9-15,17-19]. As suggested in a recent editorial, it may be reasonable to assume that most patients with ARDS need treatment in an ICU, but many patients with milder ALI may not receive care in an ICU for medical or non-medical reasons; little is known about these patients [21].

We therefore performed a prospective observational study with the following objectives: to document the association between predefined clinical conditions and the development of ALI/ARDS by using the AECC definitions; to document the frequency of ALI/ARDS development outside the ICU; and to document the temporal relationship between antecedent clinical risk conditions, admission to the ICU, and diagnosis of ALI/ARDS.

## Methods

### Ethical considerations

The ethics committee at each participating hospital approved the study and waived the need for informed consent.

### Patients

Patients were recruited from three hospitals in the south of the Comunidad de Madrid, Madrid, Spain, from 1 March to 30 June 2003. This study duration was chosen on the basis of resources available for data collection. These three general hospitals each have tertiary ICUs and residency training programs. They service adjacent, well-defined geographic areas; on the basis of 2001 census data they include a total of 573,149 individuals older than 18 years of age [22]. The usual practice in the Comunidad de Madrid is for patients to present to or be transferred to their geographically assigned hospital when acute care admission is required.

We screened patients who were admitted to an ICU or hospital ward and enrolled them if they were admitted with or developed one or more clinical conditions previously reported to be linked to the development of ARDS [3,9,19,20,23], defined by using standard definitions (see Tables 1, 2, 3 for details) [3,24-27]. Patients with pneumonia, aspiration of gastric contents, pulmonary contusion, near-drowning, or inhalational injury were grouped as pulmonary cases; others were extrapulmonary. We excluded patients who were younger than 18 years, discharged from hospital alive within 48 hours of admission, transferred from another hospital with a pre-existing diagnosis of ALI/ARDS, or previously enrolled in the study cohort. In the medical–surgical ICUs and each at-risk ward area, all admitted patients were actively screened for the presence of these clinical conditions associated with ARDS by physician co-investigators, who reviewed admission records and patient charts and liaised with nurses and physicians on each ward to identify patients with these clinical risk conditions.

**Table 1:** Baseline characteristics and clinical risk conditions: ICU and ward

Characteristic or condition	All patients	ICU admissions	Non-ICU admissions	*P*^a^
Number	815	108	707	
Age, years; median (interquartile range)	74 (55–83)	66 (48–78)	74 (56–84)	<0.001
Female sex, *n *(percentage)	450 (55.2)	61 (56.5)	389 (55.0)	0.836
McCabe score				
Non-fatal	618 (75.8)	81 (75.0)	537 (76.0)	
Ultimately fatal	178 (21.8)	25 (23.1)	153 (21.6)	0.894
Fatal	19 (2.3)	2 (1.9)	17 (2.4)	
Medical (versus surgical) admission	663 (81.3)	56 (51.9)	607 (85.9)	<0.001
Clinical risk conditions, *n *(percentage)				
Sepsis	704 (86.4)	84 (77.8)	620 (87.7)	0.005
Pneumonia	233 (28.6)	30 (27.8)	203 (28.7)	0.841
Aspiration	16 (2.0)	1 (0.9)	15 (2.1)	0.709
Trauma	21 (2.6)	3 (2.8)	18 (2.5)	0.751
Transfusions	9 (1.1)	8 (7.4)	1 (0.1)	<0.001
Pancreatitis	75 (9.2)	3 (2.8)	72 (10.2)	0.011
Pulmonary contusion	3 (0.4)	2 (1.9)	1 (0.1)	0.048
Shock	59 (7.2)	51 (47.2)	8 (1.1)	<0.001
Other	2 (0.2)	2 (1.9)	0	0.017
Any pulmonary insult	244 (29.9)	34 (31.5)	210 (29.7)	0.707
On day of clinical risk development, median (IQR) or *n *(percentage)				
SAPS II	26 (18–33)	40 (27–49)	28 (22–35)	<0.001
MAP	84 (74.8–95)	70 (56–83)	89 (77.8–100)	<0.001
GCS	15 (15–15)	15 (15–15)	15 (15–15)	0.647
PaO_2_/FiO_2_, mmHg	261.9 (221–310)	217.1 (158–323)	266.7 (238–314)	<0.001
Vasoactive drugs	40 (4.9)	40 (37.0)	0	<0.001
Mechanical ventilation	46 (5.6)	46 (42.6)	0	<0.001
Location (on ward)	730 (89.6)	23 (21.5)	707 (100)	<0.001
MODS score	1.0 (0–3)	5 (3–7)	2 (1–3)	<0.001
Days from hospital admission to insult	0 (0–1)	0 (0–5)	0 (0–0)	0.041
ALI diagnosis	20 (2.5)	9 (8.3)	11 (1.6)	<0.001
ARDS diagnosis	33 (4.0)	29 (26.8)	4 (0.6)	<0.001

ICU, intensive care unit; IQR, interquartile range; ALI, acute lung injury (PaO_2_/FiO_2 _200 to 300 mmHg); ARDS, acute respiratory distress syndrome; SAPS, simplified acute physiology score; MAP, mean arterial pressure; GCS, Glasgow coma score; PaO_2_, partial pressure of arterial oxygen; FiO_2_, fractional concentration of inspired oxygen; MODS, multiple organ dysfunction syndrome. ^a^Comparing ICU admissions with non-ICU admissions

**Table 2:** Characteristics at diagnosis of ALI/ARDS

Characteristic	ALI	ARDS	*P*
Number	20	33	
Age, years; median (interquartile range)	70.5 (42–81.8)	60.5 (46.5–79.3)	0.436
Female sex, *n *(percentage)	13 (65.0)	21 (63.6)	1
McCabe score			
Non-fatal	14 (70.0)	23 (69.7)	
Ultimately fatal	5 (25.0)	9 (27.3)	0.927
Fatal	1 (5.0)	1 (3.0)	
Medical (versus surgical) admission	13 (65.0)	20 (60.6)	0.369
Antecedent clinical risk conditions, *n *(percentage)			
Sepsis	16 (80.0)	30 (90.9)	0.405
Pneumonia	10 (50.0)	15 (45.5)	0.783
Aspiration	1 (5.0)	1 (3.0)	1
Trauma	0	0	1
Transfusions	2 (10.0)	1 (3.0)	0.549
Pancreatitis	0	1 (3.0)	1
Pulmonary contusion	0	0	1
Shock	6 (30.0)	15 (45.5)	0.386
Other	1 (5.0)	0	0.377
On day of clinical risk development, median (IQR) or *n *(percentage)			
SAPS II	37.5 (24.0–47.8)	37 (28.0–41.5)	0.604
Mean arterial pressure (mmHg)	86 (71–103)	73 (65–85)	0.076
Multiple organ dysfunction score	2 (1–3.75)	6 (4–7)	0.008
Location, percentage on ward versus ICU	12 (60)	5 (15.2)	0.002
PaO_2_/FiO_2_, mmHg	229 (210–264)	98 (78.5–146)	<0.001
Receiving mechanical ventilation, percentage	7 (35)	22 (66.7)	0.045
Tidal volume (ml)	600 (500–600)	550 (500–600)	0.615
Positive end-expiratory pressure, cmH_2_O	5 (5–8)	8 (5–10.5)	0.086
Ventilator mode, percentage volume-cycled ventilation	7 (100)	20 (91)	1
Days from clinical insult to ALI/ARDS diagnosis	0 (0–3)	2 (0–4)	0.14

ICU, intensive care unit; ALI, acute lung injury (PaO_2_/FiO_2 _200 to 300 mmHg); ARDS, acute respiratory distress syndrome; SAPS, simplified acute physiology score; PaO_2_, partial pressure of arterial oxygen; FiO_2_, fractional concentration of inspired oxygen.

**Table 3:** Outcomes by patient group

Outcome	All patients	ICU admissions	Non-ICU admissions	*P*^a^	ALI	ARDS	*P*^b^
ICU mortality	25/108 (23.1)	25/108 (23.1)	N/A		2/9 (22.2)	12/29 (41.4)	0.438
ICU length of stay	8 (4–19.5) (*n *= 104)	8 (4–19.5) (*n *= 104)	N/A		7.5 (5.3–22.5)	15 (7.5–36.5)	0.161
Duration of ventilation	7.5 (4–23) (*n *= 60)	7.5 (4–23) (*n *= 60)	N/A		6 (3–10)	17 (12–36.8)	0.035
Hospital mortality	99/815 (12.1)	29/108 (26.9)	70/707 (9.9)	<0.001	5/20 (25.0)	15/33 (45.5)	0.158
Hospital length of stay	10.0 (7–17)	21 (12–37.5)	9 (6–15)	<0.001	17 (10.0–42.0)	29 (13.3–63.8)	0.124
Cause of death							
Multiple organ failure	25 (25.3)	8 (27.6)	17 (24.3)	0.579	1 (20)	4 (26.7)	0.707
Refractory hypotension	1 (1.0)	0	1 (1.4)		0	0	
Refractory hypoxemia	4 (4.0)	1 (3.4)	3 (4.3)		0	1 (6.7)	
Arrhythmia	2 (2.0)	1 (3.4)	1 (1.4)		0	0	
Withholding-withdrawal	58 (58.6)	14 (48.3)	44 (62.9)		4 (80)	7 (46.7)	
Brain death	2 (2.0)	1 (3.4)	1 (1.4)		0	1 (6.7)	
Other	7 (7.1)	4 (13.8)	3 (4.3)		0	2 (13.3)	

ICU, intensive care unit; ALI, acute lung injury (PaO_2_/FiO_2 _200 to 300 mmHg); ARDS, acute respiratory distress syndrome; PaO_2_, partial pressure of arterial oxygen; FiO_2_, fractional concentration of inspired oxygen. ^a^Comparing ICU admissions with non-ICU admissions; ^b^comparing ALI with ARDS.

### Cohort follow-up and data collection

Enrolled patients were followed daily for the development of ALI/ARDS [3]. In addition, until the development of ALI/ARDS, we continued to screen enrolled patients daily for the development of other clinical risk conditions. Screening for ALI/ARDS diagnosis was continued for 7 days unless another clinical insult developed, in which case follow-up was continued for a total of 14 days. When a diagnosis of ALI was made we continued to follow patients daily to document potential conversion to ARDS.

At the time of enrolment we recorded demographic data, the reason for admission to hospital, previous comorbidity status (McCabe score), whether their admission was medical or surgical, their location before admission (home, other acute hospital, or chronic hospital), and the presence of comorbidities. In addition, data on each patient was collected at up to four distinct time points (if they occurred and were separated by at least 12 hours): time of clinical insult identification (enrolment); time of admission to ICU; time of endotracheal intubation; and time of development of ALI/ARDS. At each of these time points we recorded as much of the following information as was available: severity of illness (simplified acute physiology score (SAPS) II); number of organ failures and multiple organ dysfunction (MODS) score; hemodynamic data (heart rate, mean arterial pressure, central venous pressure, pulmonary artery wedge pressure, pulmonary artery pressure, and cardiac index); ventilatory data (FiO_2_, respiratory rate, ventilator mode, tidal volume, positive end-expiratory pressure, peak inspiratory pressure, and inspiratory/expiratory ratio); and arterial blood gases. All enrolled patients were followed to capture relevant outcome data, including hospital mortality and length of hospital stay, and if applicable, mortality in ICU, the length of stay in the ICU, and the duration of mechanical ventilation.

### Data coordination and quality assurance

We held monthly meetings between physicians at all centres, including the coordinating centre (Hospital Universitario de Getafe), to address issues or problems with definitions or enrolment. Case report forms were sent to the coordinating centre and were double-entered into a database. Blank fields or improbable values generated queries that were returned to each centre for correction. The coordinating centre selected a random 10% sample of surveyed patients at each hospital, and data were re-abstracted from the medical records by study personnel from one of the other hospitals to ensure validity.

### Statistical analysis

Results are expressed as medians and interquartile range, or proportions with 95% confidence intervals (CI) as appropriate. We used the Mann–Whitney *U *test to compare continuous variables, and the χ^2 ^test or Fisher's exact test to compare proportions, as appropriate. Two-tailed *P *values of less than 0.05 were used to indicate statistical significance. If patients initially presented with ALI and then went on to develop ARDS, we included them only in the ARDS group. Times to diagnosis of ALI/ARDS were compared by using a Kaplan–Meier survival analysis. All analyses were conducted with SPSS version 12.0 (SPSS Inc., Chicago, IL).

## Results

### Patients

**Figure 1:**
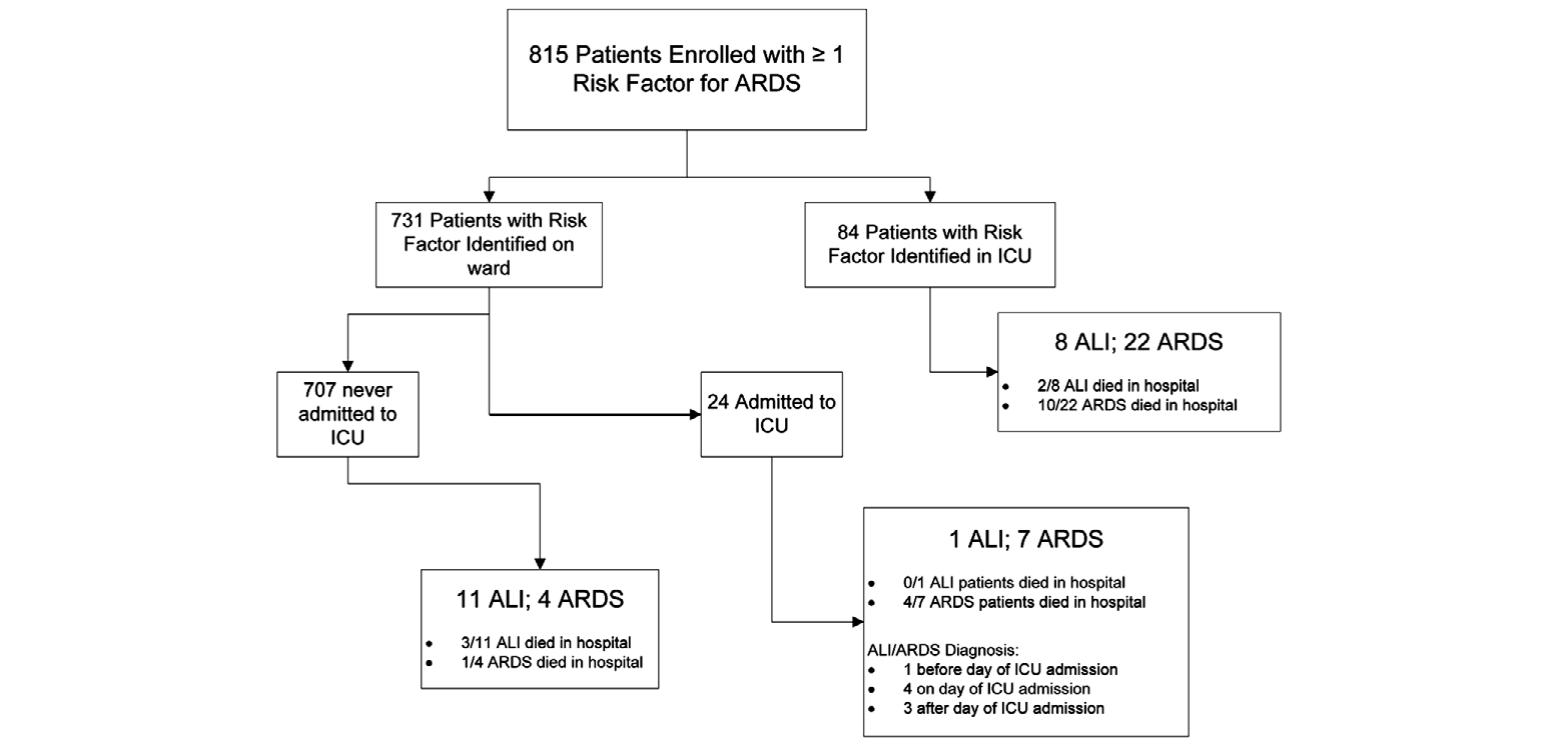
Patient flow diagram. Locations (ward versus intensive care unit) of risk factor identification and diagnosis of acute lung injury/acute respiratory distress syndrome (ALI/ARDS) are displayed along with hospital outcomes for each group. ICU, intensive care unit.

During the 4-month study period a total of 15,852 adults were admitted to the three hospitals, 815 (5.1%) of whom were enrolled after being identified with at least one clinical condition linked to ALI/ARDS; 108 (13.3%) received care in the ICU, whereas 707 (86.7%) were cared for only on another ward (Figure 1). Demographic information and data collected on the day that the clinical insult was identified are displayed in Table 1. Sepsis syndrome was the most clinical insult, seen in 86% of cases, with pneumonia being next most frequent at 29% (although these categories were not mutually exclusive).

### ALI/ARDS risk by clinical insult

A total of 53 patients (6.5%; 95% CI 5.0 to 8.4%) developed ALI/ARDS, 20 (2.4%) with ALI and 33 (4.0%) with ARDS. Three of the 33 patients with ARDS initially presented with ALI and then went on to ARDS after 1 day in two cases, and after 4 days in the third patient. These rates correspond to incidences of 27.7, 10.5, and 17.3 cases per 100,000 population per year for ALI/ARDS, ALI, and ARDS, respectively. When only ICU cases are considered these rates are lower, at 19.9, 4.7, and 15.2 cases per 100,000 population per year, respectively.

**Figure 2:**
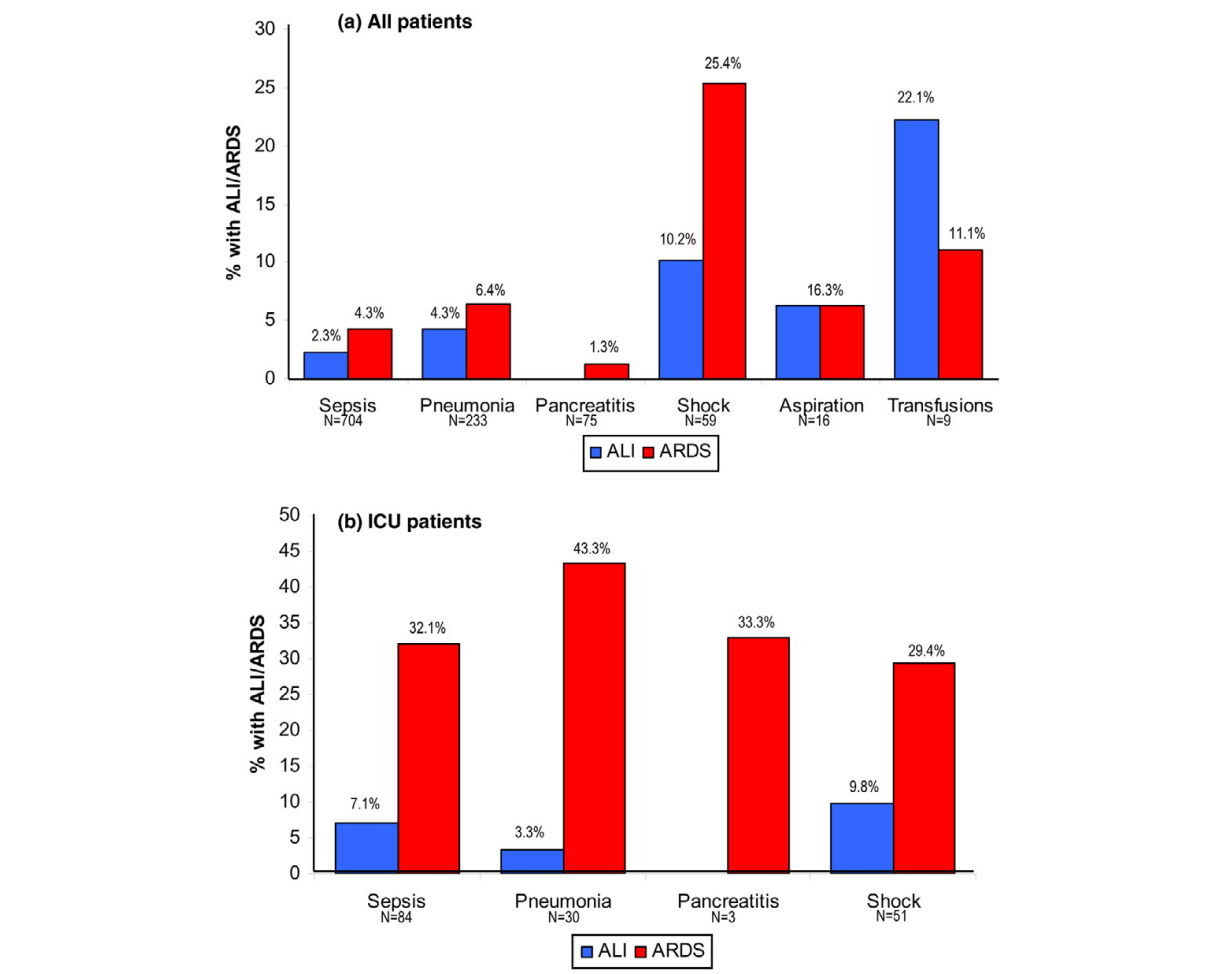
Prevalence of ALI and ARDS by clinical risk condition. The proportion of patients with each clinical risk condition who went on to develop acute lung injury (ALI; blue columns) or acute respiratory distress syndrome (ARDS; red columns) is shown for all patients **(a) **and for only those admitted to the intensive care unit **(b)**. In both panels the number of patients at risk with each clinical insult is displayed numerically below each category label.

By definition, patients with ARDS had worse hypoxemia than patients with ALI; patients with ARDS were also more likely to be diagnosed in the ICU and to be receiving mechanical ventilation at the time of diagnosis (Table 2). Figure 2a displays the proportion of patients with each antecedent clinical condition who went on to develop ALI or ARDS. The likelihood of developing ALI/ARDS was not equal between pulmonary and extrapulmonary risk conditions (37/244 (15.2%) versus 26/571 (4.6%); *P *< 0.001), nor among the mutually exclusive groupings risk conditions of shock 21/59 (35.6%), pneumonia without shock 20/211 (9.5%), and non-pulmonary sepsis without shock 6/432 (1.4%; *P *< 0.001). Examining only ICU patients (Figure 2b) leads to a much higher assessment of risk for each clinical insult. The frequency of ALI/ARDS also increased with the presence of an increasing number of clinical risk conditions (2.2%, 13.9%, and 20.8% for one, two, and three clinical conditions, respectively; *P *< 0.001).

### ALI/ARDS timelines and outcomes

**Figure 3:**
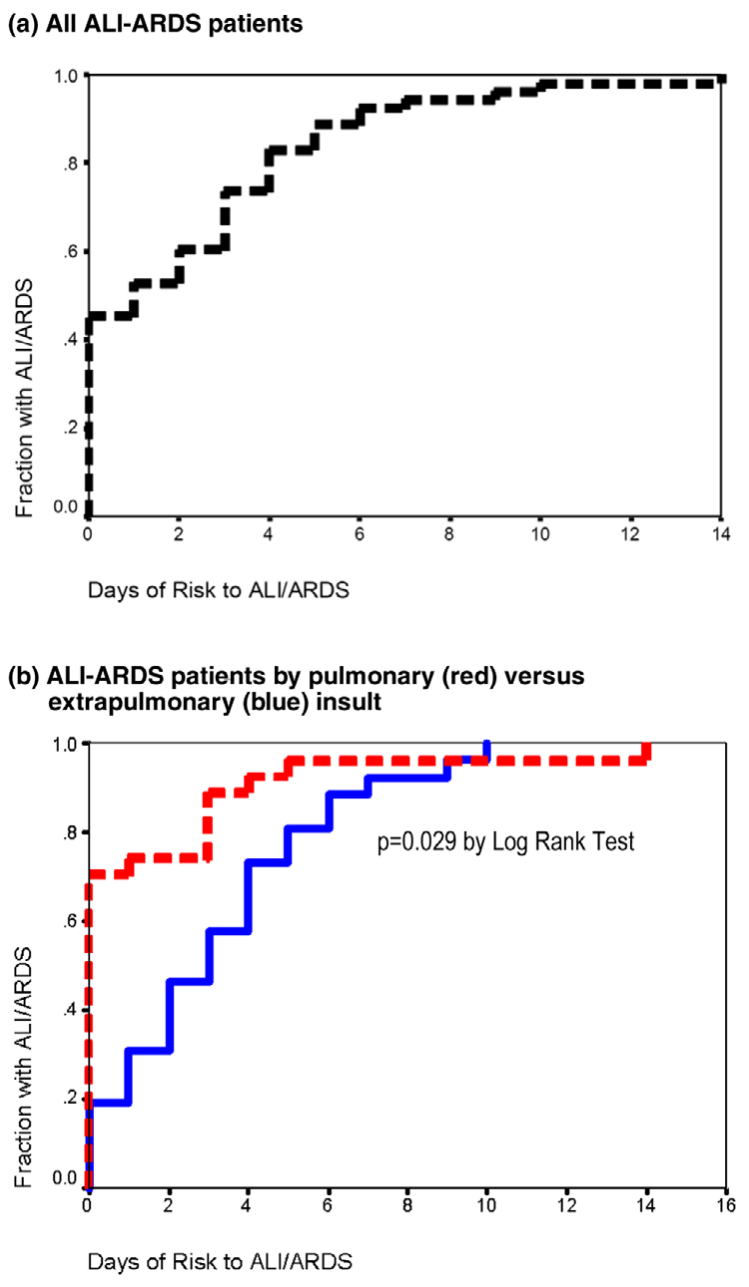
Time from clinical risk to diagnosis of ALI/ARDS. Kaplan–Meier curves displaying time from clinical risk condition to diagnosis of acute lung injury/acute respiratory distress syndrome (ALI/ARDS) are shown for all patients **(a) **and separated according to pulmonary (red line) versus extrapulmonary (blue line) risk conditions **(b)**.

A diagnosis of ALI/ARDS was made after a median of 1 day (interquartile range 0 to 4 days) from the day of clinical insult in all patients (Figure 3a), and was not statistically different between patients with ALI and those with ARDS (Table 2). In contrast, patients who developed ALI/ARDS with pulmonary conditions did so more quickly than extrapulmonary patients (median 0 (interquartile range 0 to 2) versus 3 (1 to 5) days; *P *= 0.001; Figure 3b).

**Figure 4:**
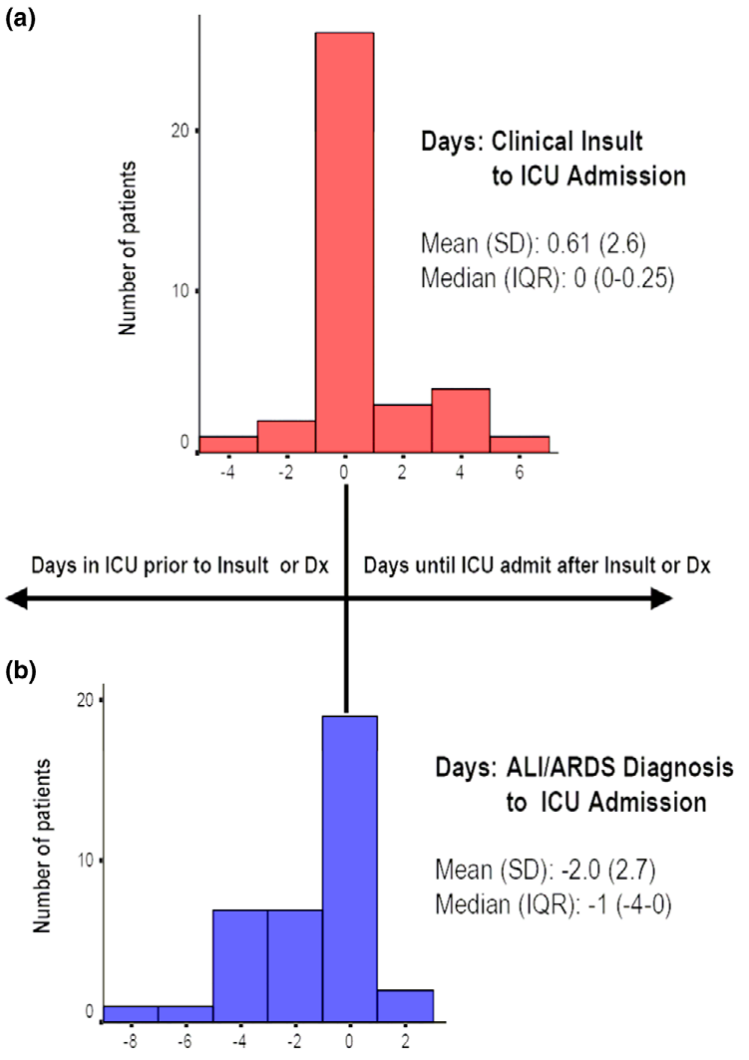
Timing of ICU admission relative to clinical risk and diagnosis of lung injury. Frequency histograms are shown for the timing of intensive care unit (ICU) admission relative to development of clinical risk condition **(a)**, and diagnosis of acute lung injury (ALI)/acute respiratory distress syndrome (ARDS) relative to ICU admission **(b)**, including together all patients with ALI and ARDS admitted to the ICU. Dx, diagnosis; IQR, interquartile range.

Patients with ARDS received ICU care significantly more frequently than patients with ALI (29/33 (88%) versus 9/20 (45%); *P *= 0.001), although the durations of ICU stay (ARDS 15 (7.5 to 36.5) versus ALI 7 (5.25 to 22.5) days; *P *= 0.16) and the times from ICU admission to diagnosis of ALI/ARDS (ARDS 1 (0 to 3.5) versus ALI 3 (0 to 4.5) days; *P *= 0.81) were not significantly different between these groups. Grouping together all patients with ALI/ARDS admitted to the ICU, Figure 4 shows the frequency histograms for the timing of ICU admission relative to development of the first clinical insult (upper panel), and diagnosis of ALI/ARDS relative to ICU admission (lower panel); on average, patients were admitted to the ICU on the day that their clinical insult developed, and they were diagnosed with ALI/ARDS 1 day later.

The relatively small number of ALI/ARDS cases makes it difficult to interpret outcome comparisons in these groups. Both ICU mortality and hospital mortality were numerically higher for patients with ARDS than for those with ALI, but these differences did not reach statistical significance (Table 3). Patients who developed either ALI or ARDS had higher hospital mortality rates than those who did not go on to develop lung injury (25.0% ALI; 45.5% ARDS; 10.3% no ALI/ARDS; *P *< 0.001). The mortality rate in patients with ALI admitted to the ICU was 22.2% (95% CI 6.3 to 54.7%); 27.3% (95% CI 9.8 to 56.6%) of patients with ALI who remained on the ward died (*P *= 0.60).

## Discussion

The main findings of this study were as follows: a significant number of patients with ALI did not receive care in an ICU; when patients outside the ICU were included, the chance of developing ALI/ARDS with a given clinical insult was substantially lower than reported previously; and the time course from clinical insult to admission to the ICU and diagnosis of ALI/ARDS is rapid, but this process may take longer for extrapulmonary ALI/ARDS.

Although many studies have reported the frequency of antecedent clinical conditions as they occur in patients who develop ALI/ARDS [9,11-15,28,29], very few have prospectively followed patients with these conditions to document the probability of developing ARDS [17-20]. These studies all used more stringent diagnostic criteria for ARDS, including more severe hypoxaemia and four-quadrant alveolar disease on a chest radiograph [19], reduced respiratory system compliance, and a pulmonary artery wedge pressure of 12 mmHg or less [18]. Because they were all conducted in the early 1980s, none of them examined current definitions for ALI or ARDS. In addition, the current definition of some clinical predispositions, notably sepsis syndrome and pneumonia, were unavailable at the time of these studies. We believe our study to be one of the first to expand surveillance outside the walls of the ICU, capturing at-risk patients on regular hospital wards. When we included all patients (ward and ICU), the risks for developing ALI and ARDS for a given clinical insult are significantly lower than reported previously. Undoubtedly this is due, at least in part, to the inclusion of patients with milder forms of these underlying conditions who seem less likely to develop ALI and ARDS. When we restrict our analysis to the ICU, we see similar rates for sepsis syndrome or shock, as Hudson and colleagues reported for their patients with septic shock (32% and 29% versus 41%) [19]. However, our rate of ARDS with pneumonia in the ICU was significantly higher than that reported by Fowler and colleagues (43% vs. 12%) [18], probably reflecting a less stringent ARDS definition and stricter ICU admission threshold in 2003 than in 1983.

Another important finding of this study is that more than half the patients with ALI (PaO_2_/FiO_2 _200 to 300 mmHg) were managed entirely outside the ICU. This has important implications for the accurate estimation of the true burden of disease in the population [7], but it also has meaning for clinicians. First, for clinicians managing patients on medical and surgical wards, it is important to realize that many patients with acute lung injury will be managed entirely outside the ICU. These patients with ALI will need different therapy from patients with cardiogenic pulmonary edema, for whom they may be mistaken. Second, for the intensivist, the question is whether these patients with ALI should be left on the ward, or whether their outcomes would be better if they received care in the ICU. In our study the death rates were not statistically different between patients with ALI who were admitted to the ICU and those who were not (22% versus 27%). Although the confidence intervals around these estimates are wide, they certainly do not suggest that these patients with ALI were kept on the floor because they were all going to do well. It is unknown whether admission to the ICU to receive therapies such as more vigorous resuscitation or non-invasive ventilation would change this outcome. We do not have a sufficient number of patients or enough information about their ward care to specifically address this question in our study. However, as many jurisdictions move to implement critical care outreach or medical emergency response teams [30,31], the fact that there may be many patients with ALI on the hospital wards should be recognized, and such teams may facilitate their further study.

We found that, on average, patients progressed quickly from development of clinical insult, to ICU admission, to diagnosis of ALI/ARDS. The finding that most cases of ARDS occur quickly after the onset of the clinical predisposition is not new [18,19]; however, we extend this knowledge in two ways. First, our finding that most patients entering the ICU do so on the day of developing their clinical insult is new, and it underscores the potential need for rapid intervention in these patients. Second, we observed a significantly longer time to ALI/ARDS development for extrapulmonary risk conditions compared with pulmonary risk conditions. This is In contrast with the findings of Hudson and colleagues, who showed fairly comparable times of ARDS onset for sepsis and aspiration [19]. This difference may be explained by the inclusion of patients with pneumonia in the sepsis category of the earlier study, and by a more liberal ARDS definition in our study, in which patients with direct lung injury already had a significant 'head start' in reaching the syndromic thresholds for ARDS. Finally, it is worth noting that relatively few patients initially diagnosed with ALI went on to develop ARDS (13%); this is a significantly lower proportion than the 55% conversion rate reported in a recent multicentre observational study [14]. The reasons for this difference are not clear; our patients with ARDS may have progressed more quickly for whatever reason, such that their movement through ALI was not captured in our once-daily screening.

Our study has several limitations. First, enrolment was limited to three hospitals in Madrid; local practice patterns (including thresholds for ICU admission) and case mix (including a lack of trauma patients, and the fact many non-ICU patients were quite elderly) may limit the generalizability of these results. Second, in this observational study we did not have a formal protocolized screening process for documenting ALI/ARDS. Chest X-rays and arterial blood gas measurements were performed when clinically indicated according to the treating physicians; we may therefore have missed some patients with ALI/ARDS, particularly on the wards in which these test are performed less frequently. Third, we enrolled patients over only a 4-month period. This has implications both in terms of missing seasonal variations in disease patterns and, importantly, in terms of the relatively small number of ALI/ARDS cases we were able to document, leading to imprecision in our point estimates, both for risk rates for different clinical conditions and mortality rates of ALI/ARDS. In addition, the accuracy of our incidence data may be questioned because of the short duration of the study and difficulties in accurately determining the population at risk (incidence denominator). Finally, we did not have the resources available to double-screen or perform other quality control measures on patients who were not already enrolled in the cohort. It is possible that we missed some patients with our defined clinical risk conditions, especially outside the ICU; however, the large number of patients at risk who were enrolled from the wards militates against this as a major flaw. In addition, our having missed patients entirely would have biased us toward underestimating the importance of ALI on the hospital wards and should not have had a large impact on our estimates of ALI/ARDS development rates and times.

## Conclusion

We have observed that the time course from clinical insult to diagnosis of lung injury was rapid, but it was longer for extrapulmonary cases. The risk of ARDS was significantly lower than reported previously when patients outside the ICU were considered, but rates in ICU patients appeared similar. A significant number of patients with ALI received care outside the ICU; whether this is ideal requires further study.

## Key messages

• A significant number of patients with ALI did not receive care in an ICU; this observation needs further study.

• When patients outside the ICU were included, the chance of developing ALI/ARDS with a given clinical insult was substantially lower than reported previously.

• The time course from clinical insult to ICU admission and diagnosis of ALI/ARDS is rapid, but this process may take longer for extrapulmonary ALI/ARDS.

## Abbreviations

AECC = American–European consensus conference; ALI = acute lung injury; ARDS = acute respiratory distress syndrome; CI = confidence interval; FiO_2 _= fractional concentration of inspired oxygen; ICU = intensive care unit; PaO_2 _= partial pressure of arterial oxygen.

## Competing interests

The authors declare that they have no competing interests.

## Authors' contributions

NDF, FFV, and AE conceived the study. All authors contributed to the study design and interpretation of the data. FFV, FG, TH, OP, AA, GG, AA, and IR participated in the acquisition of the data. NDF performed the data analysis and wrote the first draft of the manuscript, which was then revised for intellectually important content by all authors. All authors read and approved the final manuscript.
